# Health beliefs and their relation to household, necessary, and unnecessary contacts across COVID-19 epidemic waves in Taiwan

**DOI:** 10.1186/s12889-025-25340-1

**Published:** 2025-11-24

**Authors:** Mei-Hsuan Wu, Han Fu, Chieh-Yin Wu, Pau-Chung Chen, Hsien-Ho Lin, Shu-Sen Chang, Hsiao-Han Chang

**Affiliations:** 1 Precision Medicine Ph.D. Program, College of Life Sciences and Medicine, National Tsing-Hua University, Hsinchu, Taiwan; 2 Department of Infectious Disease Epidemiology and Dynamics, London School of Hygiene & Tropical Medicine, London, UK; 3 Institute of Epidemiology and Preventive Medicine, College of Public Health, National Taiwan University, Taipei, Taiwan; 4 Institute of Environmental and Occupational Health Sciences, College of Public Health, National Taiwan University, Taipei, Taiwan; 5 Department of Public Health, College of Public Health, National Taiwan University, Taipei, Taiwan; 6 Department of Environmental and Occupational Medicine, National Taiwan University Hospital and National Taiwan University College of Medicine, Taipei, Taiwan; 7 National Institute of Environmental Health Sciences, National Health Research Institutes, Miaoli, Taiwan; 8 Taiwan Public Health Association, Taipei, Taiwan; 9 Institute of Health Behaviors and Community Sciences, College of Public Health, National Taiwan University, Taipei, Taiwan; 10 Population Health Research Center, National Taiwan University, Taipei, Taiwan; 11 Institute of Bioinformatics and Structural Biology, College of Life Sciences and Medicine, National Tsing-Hua University, 101, Section 2, Kuang-Fu Road, Hsinchu, 300044 Taiwan R.O.C.

**Keywords:** Contact behaviors, Health beliefs, COVID-19, Non-household contacts, Necessary contacts, Unnecessary contacts

## Abstract

**Background:**

The COVID-19 pandemic prompted widespread preventive measures and altered individual contact behaviors. However, limited research has examined how health beliefs shape these behavioral changes.

**Methods:**

This study investigates the association between health beliefs and close contact patterns during two COVID-19 waves in Taiwan, using a longitudinal survey conducted between July 2020 and June 2021 (the 2021 summer wave, *n* = 728) and a cross-sectional survey conducted between September and November 2022 (the 2022 autumn wave, *n* = 2,793). Participants reported close contacts from the previous day, categorized as household, non-household necessary, and non-household unnecessary contacts. Generalized estimating equations and zero-inflated Poisson models were applied to assess associations.

**Results:**

The results revealed that perceived benefit from COVID-19 preventive measures and perceived severity of infection remained high since the onset of the 2021 summer wave in Taiwan. The number of non-household contacts declined during peak periods and was associated with health beliefs in distinct ways. High perceived barriers to COVID-19 preventive measures and high perceived susceptibility to infection were linked to a greater number of non-household contacts, whereas high perceived benefit from preventive measures correlated with fewer non-household contacts. Additionally, our longitudinal analysis demonstrated that health beliefs are not static but evolve over time in response to changing epidemic conditions and public awareness.

**Conclusion:**

These findings underscore the dynamic role of health risk perception in shaping contact behavior and highlight the value of distinguishing contact types to guide public health policies and modeling of disease transmission.

**Supplementary Information:**

The online version contains supplementary material available at 10.1186/s12889-025-25340-1.

## Introduction

Since the onset of the coronavirus disease 2019 (COVID-19) pandemic, governments worldwide have implemented pharmaceutical and non-pharmaceutical interventions to mitigate the spread of disease. Given that the number of close contacts is a key determinant of disease transmissibility, reducing mobility and limiting social interactions have been shown to be effective in controlling the spread of COVID-19 transmission [1–4]. In Taiwan, the government responded swiftly to the first local outbreak in May 2021 by declaring a third-level alert [5], with the implementation of nationwide mandatory social distancing measures, school closures, and work-from-home requirements to reduce community transmission [6].

Mobility data from Google, Apple, and Facebook provided valuable information about changes in human movement and were used to evaluate the effectiveness of public health policies during the pandemic [7–11]. Google mobility data separated locations into different types, such as workplaces, retail locations, and residential areas, revealing how population flow changed under various local interventions [12, 13]. However, while mobility data can capture shifts in physical presence across locations, they do not directly reveal the level of interpersonal contact or the underlying motivations driving such behaviors. These limitations highlight the need for individual-level contact data, particularly in evaluating the psychosocial and behavioral determinants of transmission risk. To address these gaps, large-scale surveys such as CoMix were conducted across multiple countries to explore factors influencing social contact rates, including age, self-reported health status, mask-wearing, vaccination, and government-imposed restrictions [14–21].

Household and non-household contacts have been analyzed separately in several studies due to their distinct nature of contact behavior and driving factors [22]. During the COVID-19 pandemic, stay-at-home policies aimed to significantly reduce community transmission through reducing non-household contact [3, 23]. However, the number of household contacts, inherently linked to household size, barely changed [1, 21]. Similarly, contacts in workplace and school primarily depend on the size and activities of the companies, schools, and classes, and these numbers typically remained stable unless stay-at-home policies were implemented [21]. In contrast, contacts in community settings were more variable due to a wide range of non-routine activities involved, such as leisure outings and family visits, and could be directly influenced by public health interventions.

Voluntary changes in preventive behaviors during the COVID-19 pandemic have been found to be associated with individuals’ attitudes and beliefs about these behaviors and COVID-19 [24–27]. The health belief model (HBM) has been used widely in previous studies to assess individuals’ willingness to engage in preventive measures in response to infectious diseases epidemics. HBM suggests that behavior change is more likely when individuals perceive a health issue as a personal threat, believe in the effectiveness of preventive actions, and view the benefits outweighing the barriers from the action to be conducted [28]. HBM provides a useful framework for understanding the factors that influence individuals’ health-related decision-making and includes four key constructs: perceived severity (seriousness of the health condition and its consequence), perceived susceptibility (likelihood of experiencing the health condition), perceived benefits (effectiveness of the actions), and perceived barriers (obstacles to taking the actions).

Studies have applied the HBM to explore individuals’ intention to receive the COVID-19 vaccine or adopt general preventive measures against the COVID-19 epidemic [29–33]. For example, a study in Egypt observed increasing trends in perceived susceptibility, severity, barrier, and benefit over the ten weeks in the early phase of the COVID-19 epidemic [34]. It concluded that adherence to preventive behaviors could be improved by enhancing perceptions of the benefits of health-protective actions through effective health communication. Other studies have focused on the application of HBM to contact behaviors. In European countries, individuals with higher perceived severity tended to reduce social contacts, while those with a higher perceived susceptibility to the COVID-19 epidemics reported more contacts [18, 21, 35]. In contrast, studies in African countries reported no significant differences in contact behaviors based on perceived susceptibility or severity [1]. Surveys from East Asia, including Japan and China, highlighted people’s high-risk perception and anxiety about infection during the early pandemic period prompted them to take preventative behaviors [36, 37]. However, it remained unclear whether and how personal health beliefs influenced contact behaviors in the context of a generally high-risk perception in Asia.

At the beginning of the COVID-19 pandemic, the level of preventative behaviors, such as voluntary mask-wearing, varied significantly between Asian and Western countries [38, 39]. Despite low daily new cases during the early stages of the pandemic [6], Taiwanese people exhibited high compliance with mandatory preventative measures implemented during the third-level alert in 2021, including mask-wearing, social distancing, and quarantine of infected individuals [40]. Taiwan launched its COVID-19 vaccination program in March 2021, initially prioritizing healthcare workers and high-risk groups, and rapidly expanded coverage throughout 2021, ultimately achieving high vaccination rates by early 2022 [5]. By the end of 2023, Taiwan experienced three main COVID-19 peaks: May 2021, May/June 2022, and September 2022 (Fig, 1(A)). With the achievement of high vaccine coverage, the availability of rapid tests, and the lower fatality risk of prevalent strains, Taiwan shifted its epidemic prevention strategy to “coexisting with the virus” in January 2022 [41]. Most social distancing measures were lifted, although the isolation of confirmed cases and mask mandates remained. In this context, changes in the contact patterns from February 2022 were likely driven by voluntary actions.

This study aimed to investigate how health beliefs influenced close-contact behaviors during the COVID-19 epidemic in Taiwan, a setting characterized by generally high perceived risk. Using data from nationwide surveys conducted during distinct epidemic waves of COVID-19, we applied HBM to assess the changes of health beliefs across different stages of the epidemic and their relationship with contact behaviors. To better inform public health policies, we separated contact types to household, non-household necessary, and non-household unnecessary contacts. The in-depth analysis of contact behavior during the epidemic setting and its associated factors provides useful information for policy planning and practice.

## Methods

### Data collection

**Fig. 1:**
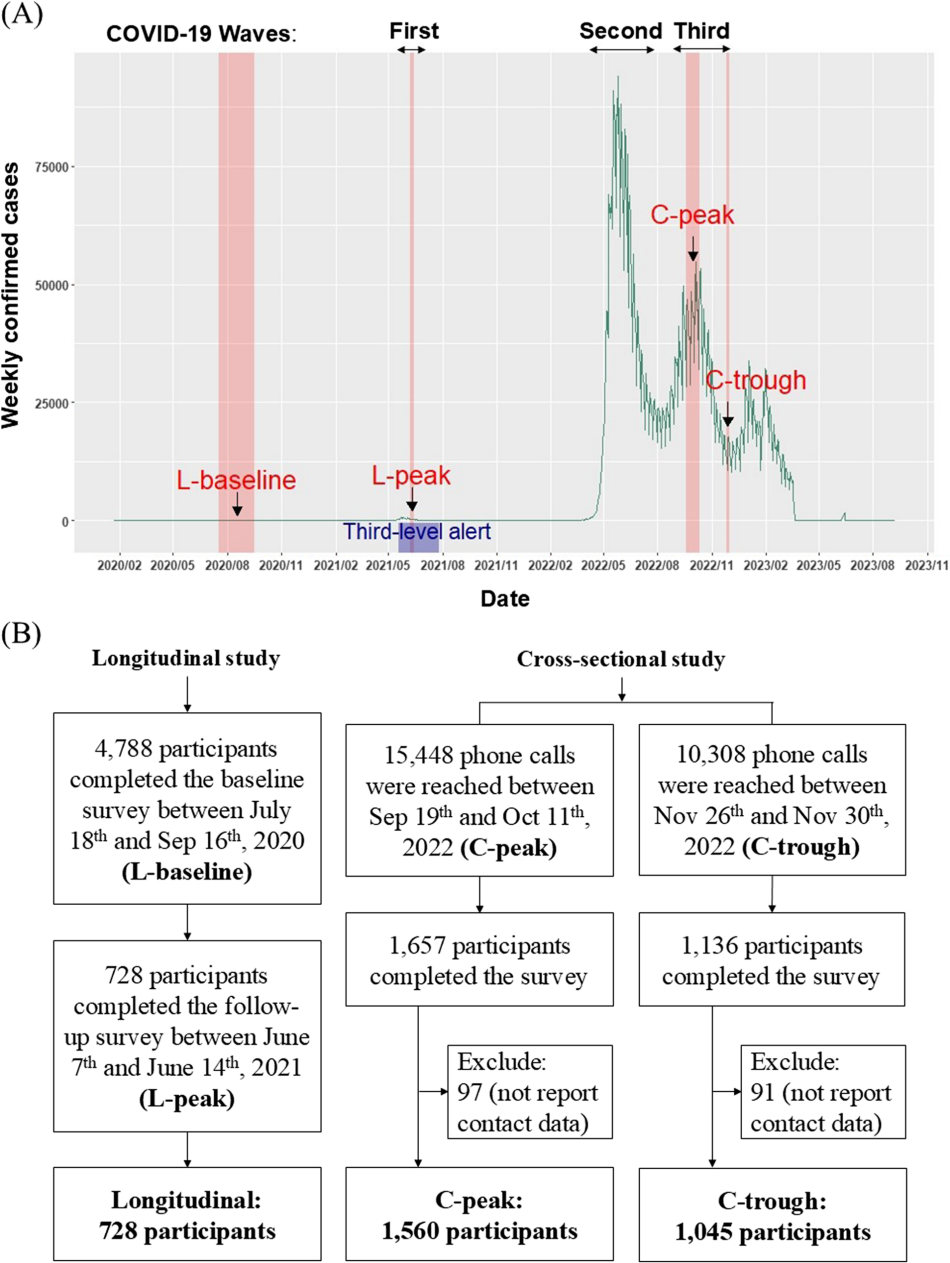
**(A)** The weekly confirmed COVID-19 cases in Taiwan from 2020 to 2023. Studying periods of the L-baseline, L-peak, C-peak, C-trough of the epidemic (marked in red) and the third-level alert period (marked in blue) were marked. (**B)** Flowchart of longitudinal (left column) and cross-sectional surveys (right column)

This paper used data from two nationwide studies among individuals aged 20 and above, residing across all 22 cities/counties in Taiwan. The first study is longitudinal, and the second is cross-sectional (Fig. 1(B)). In the first longitudinal study, the baseline data (“L-baseline”) were collected based on a face-to-face survey from July 18 to September 16, 2020, and a follow-up telephone survey (“L-peak”) was conducted in this cohort during Taiwan’s first local outbreak (2021 summer wave) from June 7 to June 14, 2021 (details described in [42]). When the baseline and follow-up surveys were conducted, the average weekly COVID-19 cases were 3 and 1,624, respectively.

In the second cross-sectional study, two separate surveys were conducted among different samples during the 2022 autumn wave: September 19 to October 11, 2022 (“C-peak”), and November 26 to 30, 2022 (“C-trough”), with average weekly cases of 300,370 and 103,095 during the two periods, respectively (Fig. 1). Due to budgetary and operational constraints, it was not feasible to conduct a follow-up survey of the original longitudinal cohort. Therefore, we used a cross-sectional design to capture the population’s current health beliefs and contact patterns during this later epidemic period. We employed a stratified sampling technique and used two types of telephone systems: landline and mobile phones. For landline calls, we systematically selected residential telephone numbers from the survey area, randomly assigning the last two digits. For mobile phone calls, we generated numbers by randomly selecting the last five digits from the mobile network user allocation database published by the National Communications Commission. The sampling ratio was designed to reflect the market shares of major telecom providers. We aimed to collect a minimum of 1,100 valid responses, with at least half obtained from landline calls and half from mobile calls. Additionally, the final sample had to meet quotas for age, gender, and geographic distribution to ensure representative coverage. During the C-peak and C-trough periods, we made 30,530 and 8,386 landline calls, reaching 8,694 and 5,964 participants, respectively. For mobile phones, we made 31,062 and 6,004 calls, reaching 6,754 and 4,344 participants. Ultimately, 1,657 and 1,136 participants completed the survey during the C-peak and C-trough periods, yielding response rates of 10.73% and 11.02%, respectively.

The surveys (details are provided in the supplementary file) included questions on demographic characteristics (e.g., age, gender, education level, residential areas, and household size), COVID-19 vaccination and infection status (for the cross-sectional study only), participants’ health beliefs related to COVID-19, and their contact behaviors on the day prior to the interview (Table S1).

### Contact behavior

We recorded the daily number of close contacts (maximum at 12). Close contact was defined as either a face-to-face conversation within 2 m or an interaction lasting more than 15 min during the day prior to the interview. For the longitudinal study, the proportion of household contacts among the total close contacts was recorded. To better understand contact behaviors, we recorded the purpose and age of each contact in the cross-sectional study and classified the contact type based on its purpose. Contacts were first categorized as household contacts and non-household contacts, with the latter being further divided into necessary contacts (including contacts for workplace, school, daily activities, hospital, and transportation) and unnecessary contacts (including contacts for leisure activities, visits to friends, family, and neighbors). The definition of the types of contacts was listed in Table 1.

**Table 1:** The definition of the types of contacts

Types	Definition
Total contacts	Total number of close contacts yesterday reported by each participant.
Household contacts	Total number of close contacts with family members living together reported by each participant on the day before the interview.
Non-household contacts	Total number of close contacts other than household members reported by each participant on the day before the interview
Necessary contacts	Number of non-household close contacts due to work, school, and daily activities.
Unnecessary contacts	Number of non-household close contacts due to leisure activities, and friends or family visits.

The primary outcome of this study was the number of daily close contacts categorized into household contacts, non-household necessary contacts, non-household unnecessary contacts. The secondary outcome was the binary indicator of zero contact based on contact types, used in the zero-inflated component of the zero-inflated Poisson (ZIP) models to assess the likelihood of having no close contacts on the previous day. These outcome measures were used to examine how different dimensions of health beliefs, demographic characteristics, and epidemic phases influenced both the number of daily contacts (quantity) and the likelihood of no contact at all (binary zero-contact outcome), across different contact settings.

### Health belief model constructs

Participants were asked to rate their agreement with the statements corresponding to the four constructs of HBM. For perceived susceptibility to COVID-19, participants were asked if they agree with the statement, “You think that you are likely to get infected with COVID-19.” For perceived severity of COVID-19 infection, the statement was, “You think that if you are infected with COVID-19, it will cause great harm to your health.” Perceived barrier to the COVID-19 preventive measures was assessed with the statement, “You think that it is difficult to implement epidemic prevention behaviors.” For perceived benefit from COVID-19 preventive measures, participants responded to the statement, “You think epidemic prevention behaviors can effectively prevent you from contracting COVID-19.” Regarding perceived barriers and benefits, the surveys did not explicitly specify preventive behaviors. As a result, these behaviors were not limited to contact behaviors but could also include measures such as wearing a mask, frequent handwashing, and maintaining social distance.

Health beliefs were assessed using a 5-point Likert scale (1 = strongly disagree, 2 = disagree, 3 = neither agree nor disagree, 4 = agree, 5 = strongly agree). Following previous studies in behavioral health research [43–45], we grouped the scale of each HBM construct (perceived susceptibility, perceived severity, barrier, and benefit) into a binary variable in statistical analysis. For example, participants who selected “agree” or “strongly agree” (4 or 5 points) were categorized as having ‘high’ perceived susceptibility, while others were grouped as having ‘low’ perceived susceptibility.

### Statistical analysis

We applied the multivariable repeated weighting (ranking) method to adjust participant data according to sex, age, and residence, aligning it with Taiwan’s population as of May 2020 and August 2022, respectively [46, 47]. To analyze changes in perceived susceptibility, severity, barriers, and benefits, we applied the McNemar test for comparisons between the L-baseline and L-peak periods, and the Chi-squared test for comparisons between the C-peak and C-trough periods.

To examine the association between HBM constructs and contact behaviors for the longitudinal and the cross-sectional study, we employed generalized estimating equations (GEE) and zero-inflated Poisson (ZIP) models, respectively. GEE was used in the longitudinal study to account for repeated measures on the same individuals. A Poisson distribution was assumed for the count outcomes (e.g., number of total, household, or non-household contacts), and a log link function was applied to estimate rate ratios (RR) and 95% confidence intervals (CI).

Given the over-dispersed nature of the contact data in the cross-sectional study, ZIP models were employed. ZIP models include two components: a logistic model for predicting the probability of zero counts and a Poisson model for predicting the count values. These models estimated the RR (Poisson part) and odds ratios (OR) (logistic part) and 95% CI for covariates, including epidemic periods (peak or trough), health beliefs, and their interactions with epidemic periods. The basic demographic variables (e.g., sex, age, education level [elementary school or below, junior high school, high school, college, graduate school and above], household size, and residential area [northern, southern, western, eastern and outlying islands of Taiwan]) were included and adjusted for in the regression models [35, 48–50]. Age was categorized into three groups: <30, 30–59, and ≥ 60 years. This grouping strategy balanced conceptual relevance and statistical stability, while avoiding finer bands that would have reduced power and increased the risk of multicollinearity. These covariates were selected based on theoretical relevance to the HBM framework and prior empirical evidence regarding determinants of contact behaviors [14, 15]. All core variables were retained in the models regardless of statistical significance, due to their conceptual importance. To reduce multicollinearity, variables with a variance inflation factor (VIF) greater than 10 were excluded.

In the ZIP model, RR > 1 from the Poisson model indicates a higher number of contacts, while OR < 1 from the logistic component of the ZIP model indicates a lower likelihood of reporting zero contacts — both reflecting an increased tendency for social interaction. We also compared models with and without interaction terms; results for the main effects were generally consistent across specifications. In rare cases where they differed, we described the main effects based on the model without interactions. All statistical analyses were performed using R version 4.2.2, and the analysis code is available on GitHub (https://github.com/hhc-lab/contact-health-belief).

## Results

Two rounds of nationwide surveys conducted during the 2021 summer wave and 2022 autumn wave of COVID-19 in Taiwan were analyzed, including both longitudinal and cross-sectional designs. Figure 1 shows the timeline of the COVID-19 pandemic in Taiwan and the epidemic periods (i.e., ‘peak’ and ‘trough’) when the two nationwide studies were conducted (A) and the flowchart of the sample collection process (B). The baseline survey of the longitudinal study in 2020 (L-baseline, pre-outbreak) comprised 4788 participants; among them, 728 participants completed the follow-up survey in 2021 summer wave (L-peak, during the first outbreak under a third-level alert). Baseline comparisons indicated that follow-up respondents were younger and more educated (Table S1).

The cross-sectional study comprised two separate surveys conducted during Taiwan’s 2022 autumn wave, including 1,657 participants during the C-peak (September 2022) and 1,136 participants during the C-trough (November 2022). Overall, the sample closely reflected the national demographic structure, with slight overrepresentation among younger adults and underrepresentation among the oldest age groups (Table S1). Weighted adjustment procedures were applied during analysis to account for these differences. It is important to note that the peak and trough periods of the individual surveys were defined by the relative numbers of COVID-19 cases. During the Omicron-variant outbreak in autumn 2022, when the cross-sectional study was conducted, the number of COVID-19 cases in Taiwan was significantly higher than during the Alpha-variant outbreak in summer 2021, when the follow-up survey of the longitudinal study took place. Consequently, the case count during the C-trough period was actually much higher than during the L-peak period.

### Changes in health beliefs during the epidemic

To investigate how health beliefs influenced contact behaviors, we first tested if health beliefs changed over time using data from the longitudinal study in 2020–2021. At the L-baseline period, 11.7%, 89.6%, 15.2%, and 95.6% of participants reported high perceived susceptibility, severity, barrier, and benefit, respectively (Table 2 and Fig. S1). During the L-peak period, under a third-level alert with the implementation of several strict social-distancing measures, the proportions with high perceived susceptibility and barrier increased to 32.2% and 21.3%, respectively (McNemar test, *p* < 0.001, Table 2). Conversely, the proportion of participants with a high level of perceived benefit declined from 95.6% to 93.9% (*p* < 0.001). Similar trends were observed with the 5-point Likert scale (Table S2).

**Table 2:** The proportion of participants with “high” levels of health beliefs during L-baseline and L-peak periods in the longitudinal study

Health beliefs	The proportion of “high” levels of health beliefs		The proportion of change of beliefs (*n* = 728)			*p*-value^1^
	L-baseline (*n* = 4,788)	L-peak (*n* = 728)	Unchanged	High to low	Low to high	
Perceived susceptibility	11.7%	32.2%	70.4%	5.2%	24.4%	< 0.001
Perceived severity	89.6%	90.7%	85.3%	6.2%	8.5%	0.122
Perceived barrier	15.2%	21.3%	74.6%	8.7%	16.8%	< 0.001
Perceived benefit	95.6%	93.9%	92.8%	5.4%	1.8%	< 0.001

^1^ McNemar test

In the cross-sectional study conducted during the 2022 autumn wave, the proportions of participants with high levels of perceived susceptibility and barrier were higher than those in the longitudinal study; in contrast, the proportions of participants with high levels of perceived severity and benefit were lower (Table 3 and Fig. S1). The increased perceived susceptibility may relate to the higher case numbers reported during the 2022 autumn wave, while the decreased perceived severity likely corresponds to Taiwan’s high vaccine coverage (> 90%) during that period. No statistical evidence for a difference in health beliefs was observed between the C-peak and C-trough periods (*p* > 0.05; Table 3 and Table S2). There was statistical evidence for weak correlations among all the four HBM constructs (Table S3), with the strongest positive correlation found between perceived severity and benefit.

**Table 3:** The proportion of participants with “high” levels of health beliefs during C-peak and C-trough periods in the cross-sectional study

Health beliefs	The proportion of “high” levels of health beliefs		*p*-value^1^
	C-peak (*n* = 1,657)	C-trough (*n* = 1,136)	
Perceived susceptibility	39.8%	36.6%	0.104
Perceived severity	65.2%	62.0%	0.092
Perceived barrier	27.1%	26.4%	0.740
Perceived benefit	89.8%	91.8%	0.084

^1^Chi-squared test

### Association between health beliefs, epidemic periods, and contact patterns

**Fig. 2:**
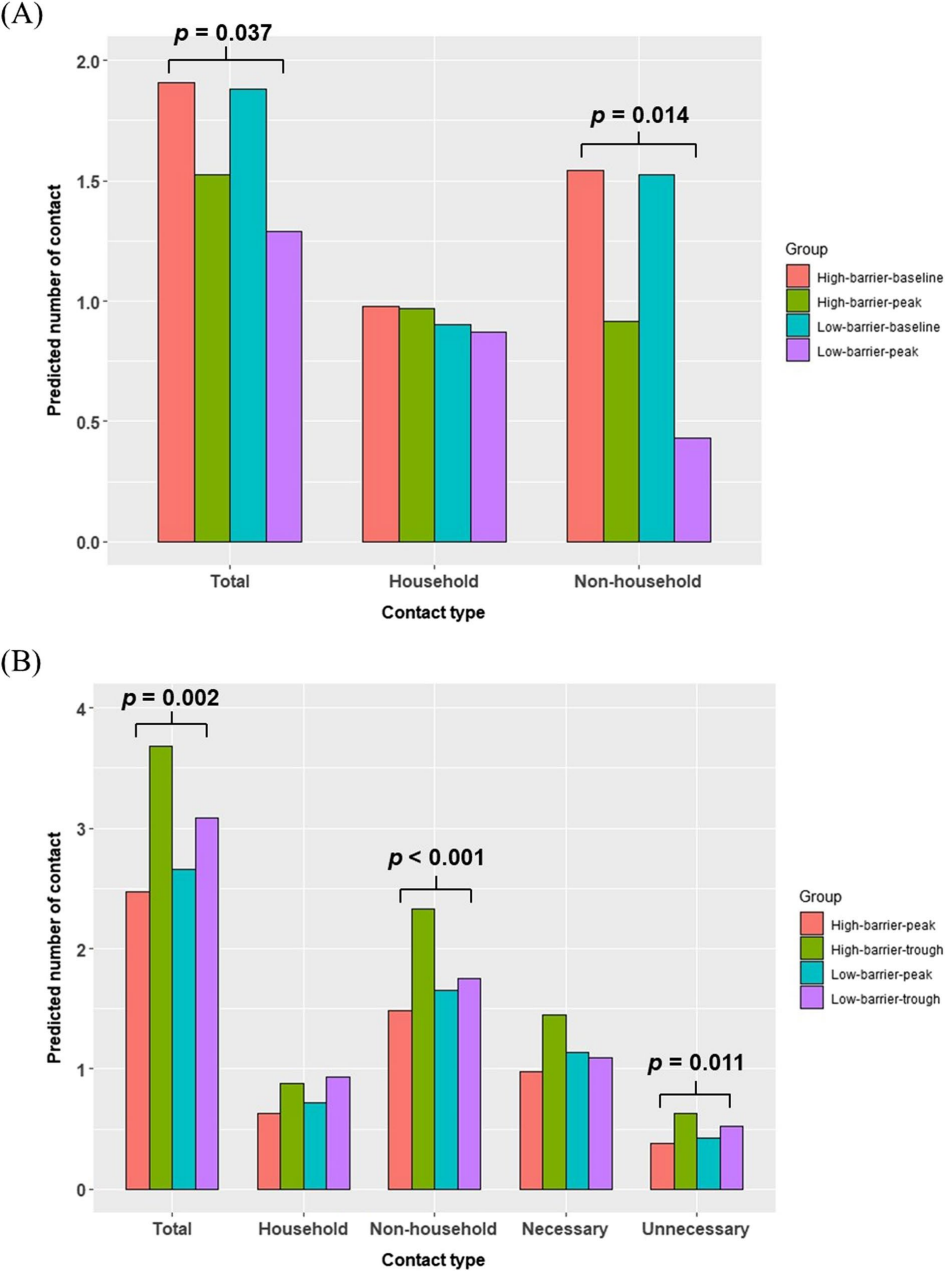
Predicted number of contacts across four groups: (1) high perceived barrier at peak period, (2) high perceived barrier at trough (baseline) period, (3) low perceived barrier at peak period, and (4) low perceived barrier at trough (baseline) period, in (**A**) the longitudinal study and (**B**) the cross-sectional study. The p-values indicate significant interaction effects between the epidemic period and perceived barrier on the predicted number of contacts. In panel (**B**), the p-values for the interaction effects were derived from the Poisson model (those from the Logistic model were not statistically significant)

In the longitudinal study, the average number of daily contacts decreased from 6.03 during the L-baseline to 3.37 during the L-peak period, with non-household contacts showing the substantial decline due to government-imposed stay-at-home policies (Table 4). Generalized estimating equations (GEE) analysis, which included demographic covariates (e.g., age, sex, education, region, and household size), revealed that epidemic periods significantly impacted the total and non-household contact numbers (both: *p* < 0.001, Table 5). In the GEE model without interaction between health beliefs and epidemic periods (Table 5), participants with higher perceived susceptibility and barrier had an increased number of total and non-household contacts, while household contacts remained similar. In contrast, high perceived severity was associated with household contacts but not with non-household contacts. When further considering the interaction effects between health beliefs and epidemic periods (Table S4), a significant interaction was observed between perceived barriers and epidemic periods (total: *p* = 0.037, non-household: *p* = 0.014). Specifically, during the peak period, participants with higher perceived barriers had an increased number of total and non-household contacts, with rate ratios (RR) [95% CI] of 1.24 [1.01, 1.51] and 1.60 [1.10, 2.31], respectively. The predicted contact number between different groups was shown in Fig. 2. In contrast, household contacts remained stable across epidemic periods (Table S4).

**Table 4:** Number of close contacts during the four periods in the longitudinal and cross-sectional studies

Contact types	Number of contacts (Mean ± SE)				*p*-value^1^
	L-baseline	L-peak	C-peak	C-trough	
Total	6.03 ± 0.06	3.37 ± 0.11	2.03 ± 0.07	2.30 ± 0.09	0.020
Household	2.27 ± 0.03	2.11 ± 0.06	0.70 ± 0.03	0.80 ± 0.04	0.076
Non-household	4.28 ± 0.06	1.46 ± 0.11	1.33 ± 0.06	1.50 ± 0.09	0.113
Necessary	NA	NA	0.96 ± 0.06	1.02 ± 0.08	0.517
Unnecessary	NA	NA	0.39 ± 0.03	0.48 ± 0.05	0.112

^1^Welch t-test were performed to test the differences between C-Peak and C-trough periods

**Table 5:** Generalized estimating equation (GEE) estimations to number of contacts of periods and high/low level of health beliefs (without interaction)

Variables	Total contact	Household contact	Non-household contact
Epidemic period: L-peak (L-baseline as ref)	0.51 [0.47, 0.55] ***	0.96 [0.88, 1.06]	0.30 [0.25, 0.35] ***
High level of susceptibility (Low level as ref)	1.12 [1.02, 1.23] *	1.04 [0.95, 1.15]	1.23 [1.05, 1.45] *
High level of severity (Low level as ref)	0.98 [0.88, 1.10]	1.19 [1.03, 1.37] *	0.91 [0.76, 1.09]
High level of barrier (Low level as ref)	1.13 [1.02, 1.25] *	1.09 [0.98, 1.21]	1.19 [1.01, 1.41] *
High level of benefit (Low level as ref)	0.98 [0.79, 1.22]	0.86 [0.71, 1.05]	1.09 [0.72, 1.66]

We also controlled for sex, age, education level, household size, and residential area in the models

The data was presented in rate ratio (RR) [95% confidence interval (CI)]

* *p* < 0.05, ** *p* < 0.01, *** *p* < 0.001

In the cross-sectional study, the number of daily contacts were consistently lower during the C-peak compared to the C-trough across all contact types, including total, household, non-household, necessary, and unnecessary contacts (Table 4). Furthermore, the cross-sectional study revealed that individuals tended to have more contact with people from their own age groups, with young people aged under 30 years reporting the highest contact rates during both the peak and trough periods (Fig. S2). In general, participants had lower number of contacts during the peak period (RRs for all contact type were less than 1, total, non-household, and unnecessary: *p* < 0.001, household: *p* = 0.011, unnecessary: *p* = 0.007; Table 6 - Poisson model), and health beliefs were associated with the number of contacts through their main effects, although the direction of impact and the specific type of contact affected varied. High perceived susceptibility and high perceived barrier were linked to an increased number of contacts, whereas high perceived benefit was associated with a reduced number of contacts.

**Table 6:** Zero-inflated Poisson (ZIP) models of number of contacts of periods and high/low level of health beliefs (without interaction)

Variables	Total contact	Household contact	Non-household contact	Necessary contact	Unnecessary contacts
Logistic model (OR [95% CI])					
Epidemic period: C-peak (C-trough as ref)	0.87 [0.73, 1.05]	0.96 [0.78, 1.18]	0.94 [0.79, 1.12]	0.79 [0.65, 0.96] *	1.19 [0.95, 1.50]
High level of susceptibility (Low level as ref)	0.70 [0.58, 0.84] ***	0.71 [0.58, 0.87] **	0.74 [0.62, 0.89] **	0.68 [0.56, 0.83] ***	0.94 [0.74, 1.19]
High level of severity (Low level as ref)	1.21 [1.00, 1.47]	1.39 [1.12, 1.72] **	0.97 [0.81, 1.16]	0.92 [0.75, 1.12]	0.97 [0.76, 1.24]
High level of barrier (Low level as ref)	1.15 [0.94, 1.42]	1.24 [0.98, 1.56]	1.02 [0.84, 1.25]	1.14 [0.91, 1.42]	0.91 [0.70, 1.18]
High level of benefit (Low level as ref)	1.09 [0.80, 1.49]	-	1.16 [0.87, 1.55]	1.13 [0.82, 1.55]	1.38 [0.97, 1.95]
Poisson model (RR [95% CI])					
Epidemic period: C-peak (C-trough as ref)	0.82 [0.77, 0.86] ***	0.86 [0.77, 0.97] *	0.82 [0.77, 0.88] ***	0.78 [0.72, 0.85] ***	0.83 [0.73, 0.95] **
High level of susceptibility (Low level as ref)	1.21 [1.14, 1.28] ***	1.14 [1.02, 1.27] *	1.23 [1.15, 1.32] ***	1.19 [1.10, 1.29] ***	1.23 [1.07, 1.41] **
High level of severity (Low level as ref)	0.99 [0.93, 1.05]	0.99 [0.88, 1.11]	0.96 [0.90, 1.03]	0.95 [0.87, 1.03]	0.87 [0.77, 1.00] *
High level of barrier (Low level as ref)	1.08 [1.01, 1.15] *	1.02 [0.89, 1.16]	1.08 [1.00, 1.16]	1.12 [1.02, 1.23] *	0.93 [0.80, 1.09]
High level of benefit (Low level as ref)	0.81 [0.74, 0.88] ***	-	0.79 [0.71, 0.87] ***	0.87 [0.77, 0.99] *	0.82 [0.68, 1.00] *

We also controlled for sex, age, education level, household size, and residential area in the models. Due to multicollinearity, the household contact model excluded the age, education level, and perceived benefit, and the unnecessary contact model excluded the education level.

The data was presented in odd ratio (OR) [95% confidence interval (CI)] for the logistic part, and rate ratio (RR) [95% CI] for the Poisson part.

* *p* < 0.05, ** *p* < 0.01, *** *p* < 0.001.

Specifically, the high perceived barrier was associated with increased total (RR = 1.08 [1.01, 1.15], *p* = 0.022) and necessary contacts (RR = 1.12 [1.02, 1.23], *p* = 0.015, Table 6 - Poisson model), showing an association between the perceived barriers to preventive measures and the behavior of limiting the number of contacts. High perceived susceptibility was associated with a greater number of contacts (RRs for all contact types were greater than 1, total, non-household, and necessary: *p* < 0.001, household: *p* = 0.021, unnecessary: *p* = 0.003; Table 6 - Poisson model) and with reduced zero-contact rates across the total, household, non-household, and necessary contacts (Table 6 - Logistic model), indicating that participants’ perceived susceptibility might have been influenced by the actual number of contacts.

High perceived severity, on the other hand, was correlated with decreased number of unnecessary contact (RR = 0.87 [0.77, 1.00], *p* = 0.049, Table 6 - Poisson model) and higher zero-household contact rates (OR = 1.39 [1.12, 1.72], *p* = 0.003, Table 6 - Logistic model). This finding indicates that participants with higher level of perceived severity reduced their leisure activities based on health considerations. High perceived benefit was associated with fewer non-household contacts (RR = 0.79 [0.71, 0.87], *p* < 0.001), including both necessary contacts (RR = 0.87 [0.77, 0.99], *p* = 0.028) and unnecessary contacts (RR = 0.82 [0.68, 1.00], *p* = 0.046). This suggests that individuals who perceived greater benefits from preventive measures were more likely to reduce non-household contacts, which may have been relatively easier to adjust through temporary lifestyle changes.

Next, we considered the interaction effects between health beliefs and epidemic periods in the ZIP models (Table S5). An interaction between perceived barriers and epidemic periods was found to have a significant impact on the numbers of total, non-household, and unnecessary contacts (Table S5 - Poisson model and Fig. 2). Interestingly, the direction of this interaction impact (RRs were less than 1) differed from findings in the longitudinal survey (RRs were greater than 1, Table S4). Specifically, individuals with higher perceived barriers were more likely to reduce their contact numbers during the peak period compared to the trough period (total: RR = 0.82 [0.72, 0.92], *p* = 0.002; non-household: RR = 0.75 [0.65, 0.88], *p* < 0.001; unnecessary: RR = 0.69 [0.52, 0.92], *p* = 0.011), while household and necessary contacts remained unaffected (*p* > 0.05, Table S5 - Poisson model, Fig. 2). Moreover, the interaction between perceived susceptibility and epidemic periods was significant for non-household contacts (RR = 1.16 [1.01, 1.33], *p* = 0.037), indicating that participants with a high level of perceived susceptibility exhibited a smaller reduction in non-household contacts during the peak period compared to those with a low level of perceived susceptibility.

## Discussion

Through the two surveys conducted during the 2021 summer and 2022 autumn waves of COVID-19 in Taiwan, our study examined changes in social contact patterns across different phases of the pandemic and their relationship with health beliefs. We found that the number of contacts was significantly lower during the peak periods of both waves. Health beliefs could influence contact behaviors in various ways: high perceived barriers and susceptibility were generally associated with an increased number of contacts, while high perceived severity and benefit were associated with a reduced number of contacts. In addition, our longitudinal survey demonstrated that health beliefs are not fixed but could change over time.

Recognizing different implications of contact types, we assessed contact behaviors by categorizing them into household, non-household necessary, and non-household unnecessary contacts. During the L-peak period of the longitudinal study, the number of contacts, especially non-household contacts, significantly decreased compared to the L-baseline period. This reduction coincided with the government-implemented third-level alert measures. Similar trends have been observed in previous studies, which reported reductions in contact numbers as the stringency index of COVID-19 restrictions increased [1, 18].

The average daily contact numbers showed a significant decrease from an average of 12.5 contacts in 2010 [51] to < 7 right before the first local COVID-19 outbreak in Taiwan (L-baseline). Despite this reduction, contact patterns among age groups remained consistent before and during the COVID-19 outbreak and were comparable to findings in European countries reported in the Comix study, where larger numbers of contacts were observed among younger groups and between individuals of similar ages [52]. Additionally, the proportion of non-household contacts among all contacts decreased from nearly 70% to 43% with the mandatory measures during the COVID-19 outbreak (L-peak). The proportion of non-household contact remained at 65% during both the C-peak and C-trough periods, when COVID-19 measures were no longer mandatory, and restrictions on contacting non-household members depended solely on voluntary actions.

Despite having relatively few cases during the first wave, the Taiwanese society exhibited a high level of perceived severity, with an average score above 4.3 (out of 5). This contrasts with European countries, where average scores ranged between 3 and 4 in most countries participating in the Comix survey during 2020–2021 [18]. Our findings also revealed that, regardless of fluctuations in COVID-19 case numbers, the proportion of individuals with higher levels of perceived severity (>89%) and benefit (>93%) remained consistently high. This suggests a heightened level of alertness and perceived severity within the Taiwanese population, possibly linked to the collective memory of the severe acute respiratory syndrome (SARS) outbreak in 2003 [51].

The number of household contacts, which naturally depends on household size and is harder to reduce during an epidemic, remained mostly stable compared to non-household contacts. This finding aligns with previous studies in Taiwan, where a significant proportion of the population voluntarily limited interactions with non-household members, particularly friends (analogous to our “unnecessary non-household contact”) and colleagues or classmates (analogous to our “necessary non-household contact”) [53]. The only significant reduction in household contacts was observed among individuals with high perceived severity during the autumn 2022 wave (Table S5 - Logistic model). This suggests that reducing household contacts was still possible when individuals perceived such interactions as a significant health threat, perhaps by staying in separate rooms or temporarily relocating elsewhere. Moreover, this period coincided with the relaxation of most epidemic prevention policies, confirmed cases and their close contacts were still required to quarantine for at least five days. Therefore, the increase in the proportion of individuals with zero household contacts among those with high perceived severity was possibly in part due to greater compliance with quarantine requirements if they or their close contacts had recently been infected.

Previous studies have also reported associations between health beliefs and contact numbers. It has been suggested that individuals with high perceived severity report fewer contacts, while those with high perceived susceptibility report more contacts [18, 21, 35]. Our findings align with these observations but provide additional insights by identifying the relative contributions of the household, non-household necessary, and non-household unnecessary contact types. In the longitudinal study, we found that participants with a high level of perceived severity reported an increase in household contacts, whereas in the cross-sectional study, those with high perceived severity had a higher proportion of zero household contacts and a lower number of unnecessary contacts. This suggests that health risk perceptions influence contact behaviors differently across waves. Similar to findings from other studies, participants with high perceived susceptibility reported a greater total number of contacts. This may partly reflect the nature of the questionnaire design in both our study and previous research, where respondents with a higher number of contacts tend to rate their susceptibility as higher.

The impact of the interaction between high perceived barriers and epidemic peak varied between the two waves, possibly related to different epidemic status in Taiwan. During the first wave, when the weekly moving average number of cases remained below 600, high perceived barriers were associated with a higher number of non-household contacts during the peak period (L-peak). In contrast, during the 2022 autumn wave, when the weekly moving average number of cases was significantly higher (reaching as many as 46,258), high perceived barriers were associated with a lower number of non-household contacts during the peak period compared to the trough period. During the 2022 autumn wave, when overall case numbers were much higher than in the first wave, people gained experience from previous waves in Taiwan and other countries and recognized the high transmissibility of the Omicron variant. As a result, individuals with a high level of perceived barriers may have been more willing to reduce their contacts during the peak period, even if they still perceived significant barriers to protective behaviors.

While this study drew from a representative sample of 3,521 participants, several limitations should be acknowledged. First, our definition of close contact was based on conversational distance (within 2 m) or interaction duration (more than 15 min), and it did not explicitly specify whether physical contact, such as handshakes or hugs, was included. This choice was made to align with the Taiwan CDC’s case definition during the COVID-19 pandemic, which focused primarily on proximity and exposure duration rather than physical touch. We also capped the number of reportable contacts at 12 per day, which was sufficient for most participants (only 4.3% and 1.2% responses in the longitudinal and cross-sectional study reached the maximum of 12). However, the reporting cap may have led to an underestimation of certain high-risk interactions, particularly in occupational or social settings involving frequent contact. Future studies can explicitly incorporate physical contact measures to enhance comparability with international surveys and can adopt methods that permit group-level reporting to better capture high-contact environments. Additionally, by focusing on close-contact behaviors within a 24-hour period, we were not able to address the variations in weekly contact patterns (e.g., weekday vs. weekend) and the contributions of non-close contacts to disease transmission. However, the 24-hour period has been applied and validated in previous contact diary studies and demonstrated a useful understanding of general contact patterns in the population [51]. Non-close contacts were likely to have a limited effect on overall transmission, and therefore our definition of close contacts, which accounted for both intensity and duration, captured the primary drivers of transmission.

Second, the survey was limited to individuals aged 20 years old and older, excluding children and teenagers, who typically have high rates of necessary contacts, particularly in school and family settings. Further research is needed to understand whether the relationship between health beliefs and contact behaviors during an epidemic is similar for children and teenagers compared to adults. Third, the definitions of perceived barriers and benefits in the questionnaires pertain to general epidemic prevention behaviors (e.g. including vaccination) rather than specifically addressing contact behaviors. Therefore, these measures should be interpreted as reflecting attitudes and thoughts about general prevention behaviors. Fourth, variability in interview skills among interviewers may have introduced some inconsistencies into the data. Nonetheless, with structured questions and standardized instructions, the inter-rater bias was minimized. Fifth, due to the observational nature of this study, the possibility of reverse causality cannot be ruled out. For instance, participants with higher household contact frequencies may have subsequently reported lower perceived severity to align with their behaviors, rather than health beliefs shaping contact behaviors. Lastly, the use of a longitudinal design for the early epidemic phase and a cross-sectional design for the later phase may limit comparability across periods. Although both samples were population-based, the absence of follow-up data for the original cohort introduces the possibility of between-group differences unrelated to epidemic timing. Panel studies that track the same individuals across multiple epidemic phases could provide more robust insights into temporal changes in health beliefs and behaviors.

Maintaining intensive preventive measures against an epidemic over an extended period is challenging, as compliance fatigue for COVID-19 measures has been reported in multiple countries [54]. While not inferring causality, our study provides the first report from Asia on changes in health beliefs and contact patterns across different epidemic phases. These findings have important implications for designing effective, phase-specific social distancing communication strategies, tailored to individuals with heterogeneous contact types and varying health beliefs.

## Conclusions

Our study provided critical insights into how health beliefs link with close-contact behaviors in Taiwan throughout COVID-19 epidemic waves. We observed that Taiwanese participants had heightened perceived severity from the onset of the epidemic, which significantly shaped their contact behavior. Perceived susceptibility, barriers and benefits significantly influenced non-household contacts, while perceived severity was inversely associated with household contacts. Additionally, differences in necessary and unnecessary contacts suggested that a more refined classification of non-household interactions could provide further insights for monitoring interventions and policy design. These findings are crucial for developing effective public health policies and interventions that consider the diverse nature of social contacts to control infectious disease outbreaks.

## Supplementary Information

Supplementary Material 1.

## Data Availability

The original raw survey data used in this study are owned by the Ministry of health and welfare and cannot be publicly shared due to legal restrictions. Derived data generated and used in this study are available from the corresponding author upon reasonable request.

## Abbreviations

COVID-19: Coronavirus disease 2019
HBM: Health belief model
L-baseline: Baseline survey in the longitudinal study
L-peak: Follow-up survey in the longitudinal study
C-peak: Peak period survey in cross-sectional study
C-trough: Trough period survey in cross-sectional study
GEE: Generalized estimating equations
ZIP: Zero-inflated Poisson model
RR: Rate ratio
OR: Odds ratio
CI: Confidence intervals
VIF: Variance inflation factor
SARS: Severe acute respiratory syndrome
