# Supplementary material for: Health beliefs and their relation to household, necessary, and unnecessary contacts across COVID-19 epidemic waves in Taiwan
Source: BMC Public Health. 2025 Nov 24;25:4117. doi: 10.1186/s12889-025-25340-1 (PMC12642176; doi:10.1186/s12889-025-25340-1)
Supplement: Supplementary file 1 — Supplementary Material 1. [file 12889_2025_25340_MOESM1_ESM.docx]

**Supplementary File**

**Health beliefs and their relation to household, necessary, and unnecessary contacts across COVID-19 epidemic waves in Taiwan**

**Survey questions in the longitudinal study**

*(Note: The first six questions were not included in the follow-up survey. Not all questions are reported in the present study.)*

1. Do you agree to participate in the survey?
2. What is your gender?
3. How old are you?
4. Which city do you live in?
5. What is your education level?
6. What is your occupation?
7. Do you think that you are likely to be infected with the coronavirus?
     Strongly disagree   Disagree   Neither agree nor disagree   Agree   Strongly agree   Prefer not to answer
8. Do you think that being infected with COVID-19 will cause significant harm to your body?
     Strongly disagree   Disagree   Neither agree nor disagree   Agree   Strongly agree   Prefer not to answer
9. Do you think that epidemic prevention behaviors can effectively prevent infection with COVID-19?
     Strongly disagree   Disagree   Neither agree nor disagree   Agree   Strongly agree   Prefer not to answer
10. Do you think it is difficult to implement epidemic prevention behaviors?
      Strongly disagree   Disagree   Neither agree nor disagree   Agree   Strongly agree   Prefer not to answer
11. Please recall how many people you had face-to-face conversations with or had close contact with (for more than 15 minutes at less than 2 meters distance) yesterday. (Maximum: 12)
12. Of those people, how many live with you?
13. How many people are there in your household, including yourself?
14. How many household members are 18 years old or younger?
15. How many household members are 65 years old or older?

**Survey questions in the cross-sectional study**

*(Note: Not all questions are reported in the present study.)*

1. Do you agree to participate in the survey?
2. What is your gender?
3. How old are you?
4. Which city do you live in?
5. What is your education level?
6. What is your occupation?
7. Do you think that you are likely to be infected with the coronavirus?
     Strongly disagree   Disagree   Neither agree nor disagree   Agree   Strongly agree   Prefer not to answer
8. Do you think that being infected with COVID-19 will cause significant harm to your body?
     Strongly disagree   Disagree   Neither agree nor disagree   Agree   Strongly agree   Prefer not to answer
9. Do you think that epidemic prevention behaviors can effectively prevent infection with COVID-19?
     Strongly disagree   Disagree   Neither agree nor disagree   Agree   Strongly agree   Prefer not to answer
10. Do you think it is difficult to implement epidemic prevention behaviors?
      Strongly disagree   Disagree   Neither agree nor disagree   Agree   Strongly agree   Prefer not to answer
11. Please recall who you had face-to-face contact (within 2 meters) or close contact for more than 15 minutes with yesterday. For each person, provide:
    - Age group (e.g., 20–24, 25–29, …)
    - Reason for contact (e.g., household, work, school, daily activity, transportation, hospital, neighbors, friends, relatives, leisure, others)
    - Location (indoor, outdoor)
    - Mask worn (yes, no)

| No. | Age Group | Reason for Contact | Location | Wearing a Mask |
| --- | --- | --- | --- | --- |
| 1 |  |  |  |  |
| 2 |  |  |  |  |
| 3 |  |  |  |  |
| … |  |  |  |  |

1. Have you received the COVID-19 vaccine?
     Yes, received three or more doses
     Yes, received two doses
     Yes, received one dose
     No, not yet received
2. Have you experienced any of the following due to the COVID-19 pandemic?
     Confirmed COVID-19 infection (date: ___ year ___ month ___ day)
     Isolation (due to contact tracing)
     Entry quarantine
     Self-health management / self-prevention
     Household member infected
     None of the above
3. How many people are there in your household, including yourself?
4. How many household members are 18 years old or younger?
5. How many household members are 65 years old or older?

**Supplementary Figures**

**
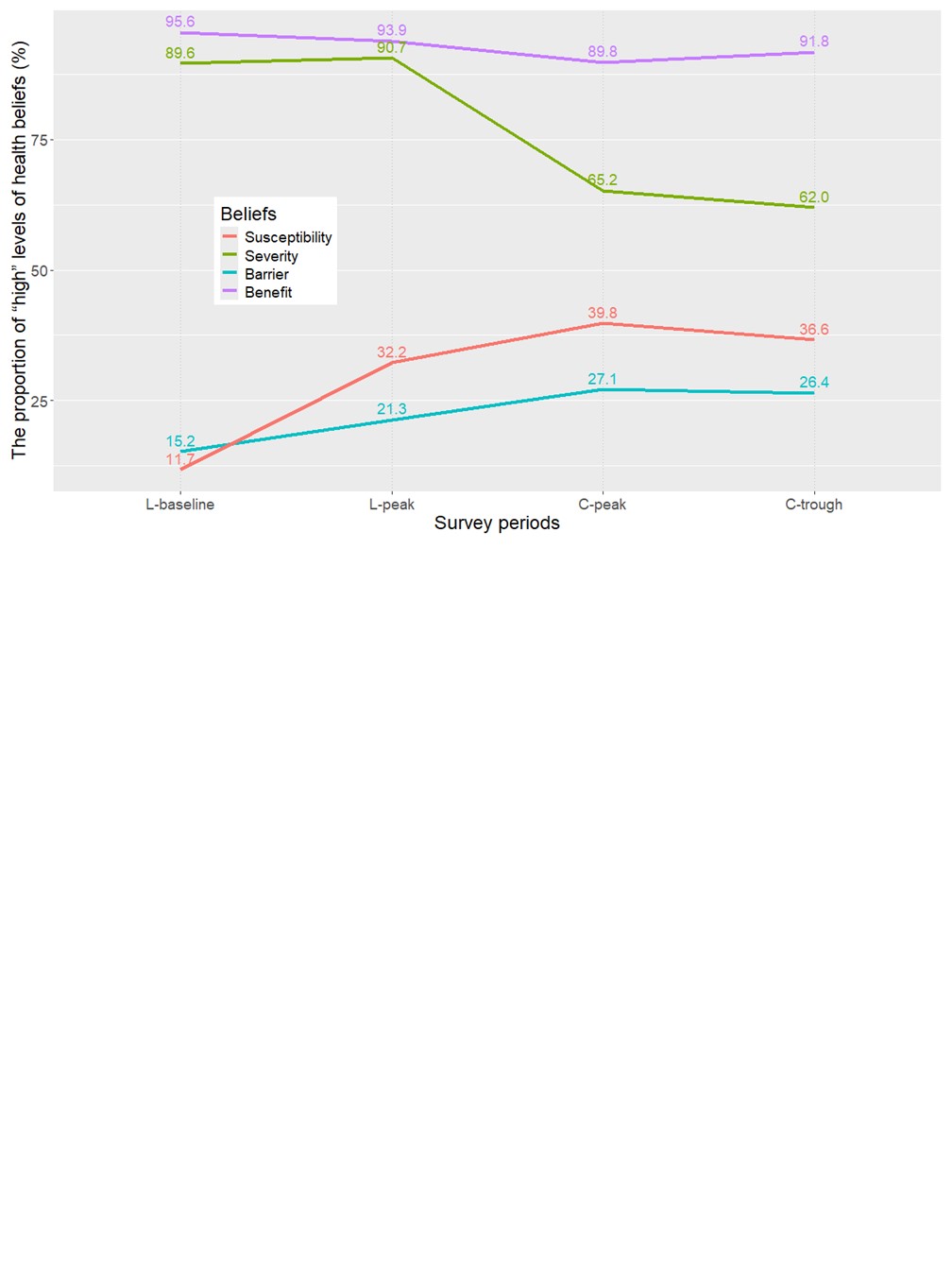
**

Fig. S1. The proportion of participants with “high” levels of health beliefs during the four periods in the longitudinal survey and cross-sectional studies

**
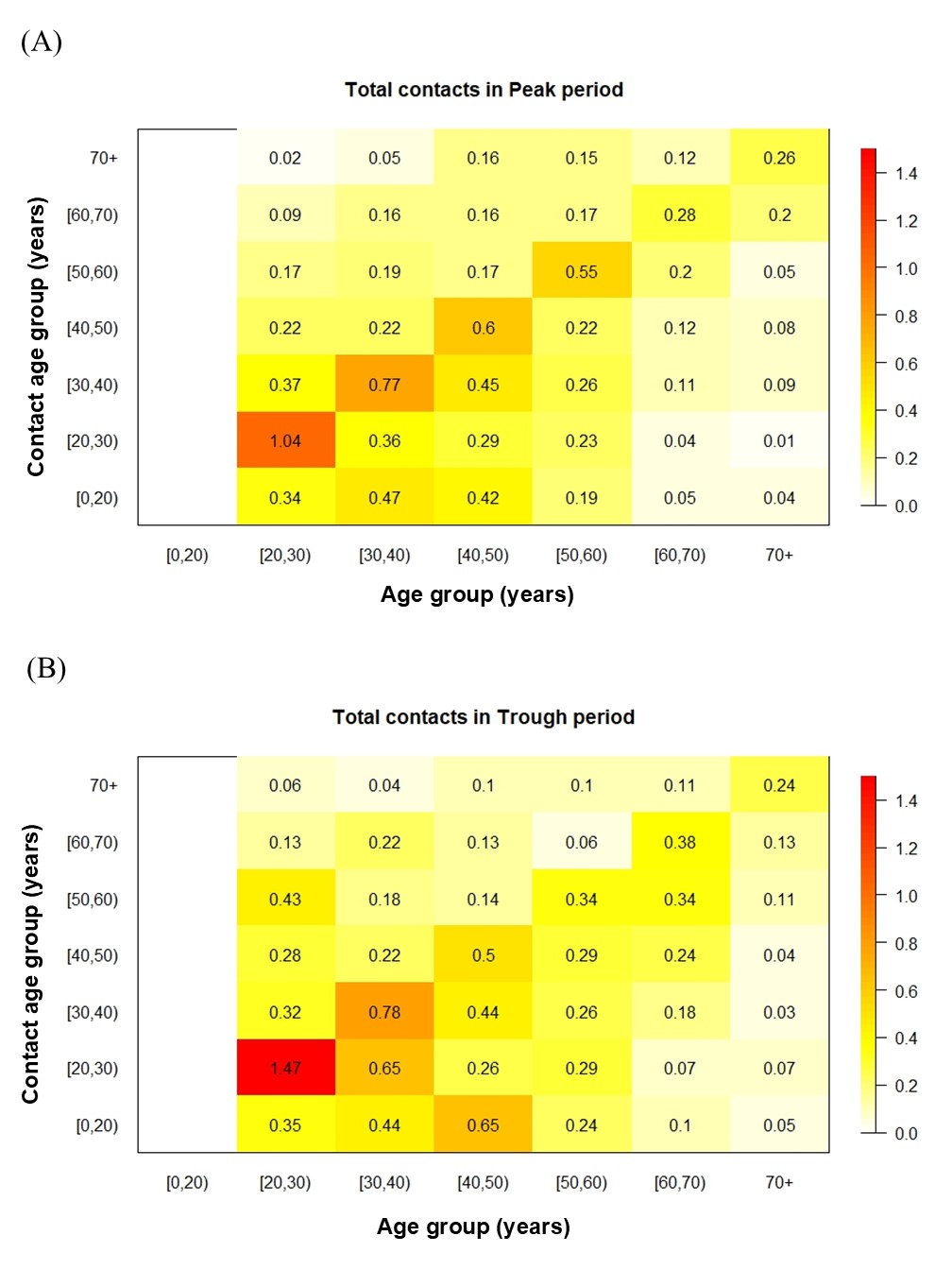
**

Fig. S2. The contact matrices in (A) C-peak period and (B) C-trough period.

**Supplementary Tables**

Our study utilized nationally representative samples of adults aged 20 and above across all regions of Taiwan. In the 2020 longitudinal baseline survey (n=4,788), the sample closely mirrored the national distribution in sex, age, and region. Female participants comprised 55.6% and males 44.4%, aligning with national demographics. The largest age groups were 20–24 (11.0%) and 40–44 (10.0%). Geographically, participants were distributed across northern (39.4%), central (23.7%), southern (34.0%), and eastern Taiwan (3.2%), though outlying islands were not represented. Over half of the participants (58.8%) reported having a college degree or higher, suggesting relatively high educational attainment.

In the 2022 cross-sectional surveys (n=1,657 in peak; n=1,136 in trough), sample distributions were also broadly comparable to national benchmarks. For example, males accounted for 48.4% and 48.8% during the peak and trough surveys, respectively. Age distributions closely approximated national proportions across all decade-based age groups. Regional coverage similarly matched national statistics: northern Taiwan accounted for 44.1% and 49.7% of participants during the peak and trough surveys, respectively, followed by central and southern Taiwan. While eastern and outlying island regions had fewer participants, they were proportionally represented given their smaller population sizes. Overall, the demographic composition supports the generalizability of our findings to the adult population in Taiwan.

Table S1. The demographic information of the longitudinal and cross-sectional studies.

| Variables | levels | Taiwan Population in 2020 | Longitudinal survey | | Taiwan Population in 2022 | Cross-sectional survey | |
| --- | --- | --- | --- | --- | --- | --- | --- |
|  |  |  | Baseline | Follow-up |  | C-peak | C-trough |
|  |  |  | (n=4788) | (n=728) |  | (n=1657) | (n=1136) |
| Sex | Male | 49.0% | 2126 (44.4%) | 298 (40.9%) | 48.9% | 801 (48.3%) | 554 (48.8%) |
|  | Female | 51.0% | 2661 (55.6%) | 430 (59.1%) | 51.1% | 856 (51.7%) | 582 (51.2%) |
| Age | 20-24 | 7.7% | 526 (11.0%) | 137 (18.8%) | 6.9% | 110 (6.6%) | 83 (7.3%) |
|  | 25-29 | 8.2% | 340 (7.1%) | 86 (11.8%) | 8.2% | 137 (8.3%) | 73 (6.4%) |
|  | 30-34 | 8.1% | 323 (6.7%) | 81 (11.1%) | 8.2% | 131 (7.9%) | 97 (8.5%) |
|  | 35-39 | 9.7% | 472 (9.9%) | 96 (13.2%) | 8.6% | 147 (8.9%) | 102 (9.0%) |
|  | 40-44 | 10.4% | 479 (10.0%) | 78 (10.7%) | 10.4% | 162 (9.8%) | 108 (9.5%) |
|  | 45-49 | 9.1% | 415 (8.7%) | 55 (7.6%) | 9.4% | 163 (9.8%) | 103 (9.1%) |
|  | 50-54 | 9.3% | 467 (9.6%) | 55 (7.6%) | 9.1% | 184 (11.1%) | 129 (11.4%) |
|  | 55-59 | 9.4% | 434 (9.1%) | 25 (3.4%) | 9.2% | 164 (9.9%) | 101 (8.9%) |
|  | 60-64 | 8.6% | 416 (8.7%) | 9 (1.2%) | 8.8% | 153 (9.2%) | 102 (9.0%) |
|  | 65-69 | 7.4% | 371 (7.7%) | 45 (6.2%) | 7.7% | 121 (7.3%) | 107 (9.4%) |
|  | 70-74 | 4.6% | 275 (5.7%) | 32 (4.4%) | 5.8% | 112 (6.8%) | 80 (7.0%) |
|  | > 75 | 7.4% | 270 (5.6%) | 29 (4.0%) | 7.6% | 73 (4.4%) | 51 (4.5%) |
| Education | Elementary |  | 386 (8.1%) | 19 (2.6%) |  | 90 (5.4%) | 61 (5.4%) |
|  | Junior |  | 406 (8.5%) | 22 (3.0%) |  | 113 (6.8%) | 93 (8.2%) |
|  | Senior |  | 1172 (24.5%) | 86 (11.8%) |  | 425 (25.6%) | 264 (23.3%) |
|  | College |  | 2310 (48.2%) | 450 (61.8%) |  | 817 (49.3%) | 599 (52.9%) |
|  | Institute |  | 509 (10.6%) | 151 (20.7%) |  | 175 (10.6%) | 116 (10.2%) |
| Residential area | North | 57.2% | 1684 (39.3%) | 287 (39.4%) | 56.2% | 731 (44.1%) | 564 (49.6%) |
|  | Central | 15.7% | 1016 (23.7%) | 340 (23.4%) | 15.6% | 370 (22.3%) | 241 (21.2%) |
|  | South | 24.4% | 1449 (33.8%) | 507 (34.8%) | 24.0% | 462 (27.9%) | 285 (25.1%) |
|  | East | 2.1% | 139 (3.2%) | 36 (2.5%) | 2.0% | 69 (4.2%) | 34 (3.0%) |
|  | Outlying Island | 0.6% | 0 (0%) | 0 (0%) | 0.6% | 25 (1.5%) | 12 (1.1%) |
| Household size | - |  | 3.75 ± 0.03 | 3.62 ± 0.06 |  | 2.84 ± 0.05 | 2.96 ± 0.06 |
| Vaccination | Unvaccinated |  | NA | NA |  | 90 (5.4%) | 41 (3.6%) |
|  | Vaccinated |  | NA | NA |  | 1565 (94.6%) | 1093 (96.3%) |
| Infection | Non-infected |  | NA | NA |  | 1195 (72.3%) | 755 (66.5%) |
|  | Ever infected |  | NA | NA |  | 456 (27.7%) | 381 (33.5%) |

Table S2. The average score of perceived severity, susceptibility, barrier, and benefit during four periods (5-point Likert scale)

| Health beliefs | Longitudinal survey | | Cross-sectional survey | |
| --- | --- | --- | --- | --- |
|  | L-baseline | L-peak | C-peak | C-trough |
| Perceived susceptibility | 2.21 ± 0.01 | 2.99 ± 0.04 | 2.84 ± 0.03 | 2.72 ± 0.04 |
| Perceived severity | 4.31 ± 0.01 | 4.46 ± 0.03 | 3.58 ± 0.03 | 3.47 ± 0.04 |
| Perceived barrier | 2.34 ± 0.01 | 2.56 ± 0.04 | 2.46 ± 0.03 | 2.41 ± 0.04 |
| Perceived benefit | 4.48 ± 0.01 | 4.46 ± 0.02 | 4.26 ± 0.02 | 4.31 ± 0.03 |

Table S3. The correlation between four aspects of health beliefs.

|  | Perceived susceptibility | Perceived severity | Perceived barrier |
| --- | --- | --- | --- |
| All | | | |
| Perceived susceptibility | 1 |  |  |
| Perceived severity | 0.054 *** | 1 |  |
| Perceived barrier | 0.139 *** | 0.031 | 1 |
| Perceived benefit | -0.079 *** | 0.197 *** | -0.177 *** |
| Longitudinal study | | | |
| Perceived susceptibility | 1 |  |  |
| Perceived severity | 0.101 *** | 1 |  |
| Perceived barrier | 0.259 *** | -0.004 | 1 |
| Perceived benefit | -0.088 ** | 0.220 *** | -0.182 *** |
| Cross-sectional study | | | |
| Perceived susceptibility | 1 |  |  |
| Perceived severity | 0.065 ** | 1 |  |
| Perceived barrier | 0.086 *** | 0.032 | 1 |
| Perceived benefit | -0.065 *** | 0.151*** | -0.179 *** |

* *p* < 0.05, ** *p* < 0.01, *** *p* < 0.001

Table S4. Generalized estimating equation (GEE) estimations to number of contacts of periods and high/low level of health beliefs

| Variables | Total contact | Household contact | Non-household contact |
| --- | --- | --- | --- |
| Epidemic period: L-peak (L-baseline as ref) | 0.47 [0.43, 0.52] *** | 0.96 [0.86, 1.07] | 0.24 [0.19, 0.29] *** |
| High level of susceptibility (Low level as ref) | 1.03 [0.90, 1.18] | 1.04 [0.85, 1.25] | 1.08 [0.88, 1.33] |
| High level of severity (Low level as ref) | 0.98 [0.88, 1.10] | 1.19 [1.03, 1.37] * | 0.91 [0.76, 1.09] |
| High level of barrier (Low level as ref) | 1.03 [0.89, 1.17] | 1.08 [0.90, 1.29] | 1.01 [0.83, 1.24] |
| High level of benefit (Low level as ref) | 0.98 [0.80, 1.22] | 0.86 [0.71, 1.05] | 1.09 [0.72, 1.65] |
| Interaction: Perceived susceptibility × Epidemic period | 1.18 [0.97, 1.42] | 1.01 [0.81, 1.27] | 1.40 [0.98, 2.00] |
| Interaction: Perceived barrier × Epidemic period | 1.24 [1.01, 1.51] * | 1.02 [0.82, 1.27] | 1.60 [1.10, 2.31] * |

We also controlled for sex, age, education level, household size, and residential area in the models. Interaction between perceived severity/ benefit and epidemic period were excluded from the models due to multicollinearity.

The data was presented in rate ratio (RR) [95% confidence interval (CI)].

* *p* < 0.05, ** *p* < 0.01, *** *p* < 0.001

Table S5. Zero-inflated Poisson (ZIP) models of number of contacts of periods and high/low level of health beliefs

| Variables | Total contact | Household contact | Non-household contact | Necessary contact | Unnecessary contacts |
| --- | --- | --- | --- | --- | --- |
| Logistic model (OR [95% CI]) | | | | | |
| Epidemic period: C-peak (C-trough as ref) | 0.82 [0.58, 1.15] | 1.10 [0.75, 1.61] | 0.78 [0.56, 1.08] | 0.70 [0.48, 1.01] | 0.91 [0.59, 1.41] |
| High level of susceptibility (Low level as ref) | 0.61 [0.45, 0.81] ** | 0.74 [0.54, 1.02] | 0.67 [0.50, 0.88] ** | 0.65 [0.48, 0.89] ** | 0.84 [0.59, 1.20] |
| High level of severity (Low level as ref) | 1.29 [0.96, 1.74] | 1.55 [1.12, 2.15] ** | 0.92 [0.69, 1.23] | 0.94 [0.68, 1.30] | 0.81 [0.56, 1.18] |
| High level of barrier (Low level as ref) | 1.03 [0.75, 1.42] | 1.18 [0.83, 1.69] | 0.90 [0.66, 1.23] | 0.87 [0.61, 1.24] | 0.95 [0.64, 1.42] |
| High level of benefit (Low level as ref) | 1.11 [0.81, 1.51] | - | 1.17 [0.87, 1.56] | 1.14 [0.83, 1.57] | 1.36 [0.96, 1.94] |
| Interaction: Perceived susceptibility × Epidemic period | 1.27 [0.87, 1.87] | 0.92 [0.61, 1.40] | 1.21 [0.84, 1.73] | 1.08 [0.72, 1.60] | 1.24 [0.77, 1.99] |
| Interaction: Perceived severity × Epidemic period | 0.90 [0.61, 1.33] | 0.83 [0.54, 1.28] | 1.09 [0.75, 1.58] | 0.97 [0.64, 1.46] | 1.38 [0.84, 2.25] |
| Interaction: Perceived barrier × Epidemic period | 1.17 [0.78, 1.78] | 1.08 [0.67, 1.72] | 1.21 [0.81, 1.81] | 1.52 [0.97, 2.38] | 0.91 [0.54, 1.54] |
| Poisson model (RR [95% CI]) | | | | | |
| Epidemic period: C-peak (C-trough as ref) | 0.81 [0.73, 0.91] *** | 0.81 [0.66, 0.99] * | 0.81 [0.71, 0.93] ** | 0.80 [0.68, 0.94] ** | 0.75 [0.59, 0.96] * |
| High level of susceptibility (Low level as ref) | 1.16 [1.07, 1.26] *** | 1.21 [1.03, 1.43] * | 1.15 [1.04, 1.27] ** | 1.13 [1.00, 1.28] | 1.22 [1.00, 1.48] * |
| High level of severity (Low level as ref) | 0.98 [0.90, 1.07] | 0.88 [0.74, 1.04] | 0.95 [0.85, 1.06] | 0.99 [0.87, 1.13] | 0.74 [0.61, 0.90] ** |
| High level of barrier (Low level as ref) | 1.20 [1.10, 1.32] *** | 1.03 [0.85, 1.26] | 1.25 [1.11, 1.39] *** | 1.20 [1.05, 1.38] ** | 1.14 [0.93, 1.40] |
| High level of benefit (Low level as ref) | 0.80 [0.74, 0.87] *** | - | 0.77 [0.70, 0.85] *** | 0.87 [0.77, 0.98] * | 0.80 [0.66, 0.97] * |
| Interaction: Perceived susceptibility × Epidemic period | 1.09 [0.98, 1.22] | 0.9 [0.72, 1.13] | 1.16 [1.01, 1.33] * | 1.11 [0.94, 1.31] | 1.08 [0.84, 1.40] |
| Interaction: Perceived severity × Epidemic period | 1.02 [0.91, 1.15] | 1.23 [0.98, 1.56] | 1.02 [0.88, 1.18] | 0.93 [0.78, 1.11] | 1.31 [1.00, 1.71] |
| Interaction: Perceived barrier × Epidemic period | 0.82 [0.72, 0.92] ** | 0.98 [0.75, 1.28] | 0.75 [0.65, 0.88] *** | 0.88 [0.73, 1.06] | 0.69 [0.52, 0.92] * |

We also controlled for sex, age, education level, household size, and residential area in the models. Interaction between perceived benefit and epidemic period was excluded from the model due to multicollinearity. In addition, the household contact model excluded the age, education level, and perceived benefit, and the unnecessary contact model excluded the education level.

The data was presented in odd ratio (OR) [95% confidence interval (CI)] for the logistic part, and rate ratio (RR) [95% CI] for the Poisson part.

* *p* < 0.05, ** *p* < 0.01, *** *p* < 0.001
